# On the Treatment of Diabetes with Secretin

**Published:** 1906-09

**Authors:** J. R. Charles

**Affiliations:** Assistant-Physician to the Bristol Royal Infirmary


### ON THE TREATMENT OF DIABETES WITH SECRETIN.

BY

J. R. CHARLES, M.A., M.D.Cantab., M.R.C.P.,
*Assistant-Physician to the Bristol Royal Infirmary.*

BAYLISS and Starling have demonstrated that when hydrochloric acid comes into contact with the epithelial cells of the duodenum and upper part of the jejunum, a substance is found therein to which they gave the name secretin. This substance is carried by the blood stream to the pancreas, on the external secretion of which gland it acts as a specific stimulant. The secretin is only formed in health, when free hydrochloric acid is passing along the duodenum, which only occurs in the presence of food.

The idea has been promulgated that it is possible, since secretin acts as a specific stimulus to the visible external secretion of the pancreas, that it may also act as a similar stimulus to the assumed internal secretion. By many it has been supposed that the internal secretion is formed in the cell-islets of Langerhans, but this appears to be by no means certain. It has been shown by Dale, who believes that these islets are derived from the ordinary alveolar cells, that there is every transitional stage between the alveolar cells and the islets. Moreover, he has shown that the proportion of islet tissue can be much increased by prolonged stimulation of the gland by secretin, and he regards the islets as cells in a state of exhaustion. If, therefore, there is only one type of secreting cell in the pancreas to elaborate both the external and internal secretions, it would appear to be not unreasonable to presume that both secretions might be stimulated by the same excitant.

A paper on the treatment of diabetes by acid extract of duodenal mucous membrane was recently published in the

*Biochemical Journal* by Moore, Edie, and Abram. As these authors point out, even if it be granted that the duodenum yields a chemical excitant for the internal secretion of the pancreas, and that in the absence of this internal secretion glycosuria occurs, then there are three places at which a breakdown may occur, resulting in diabetes. Firstly, at the duodenum, on account of the non-secretion of the excitant; secondly, at the pancreas; thirdly, in the oxidising tissues, such as the liver and muscles. Perhaps also it might be added that in some cases a defective supply of hydrochloric acid might be the cause of the failure of the elaboration of the secretin.

It must not, therefore, be supposed that all cases of diabetes will be benefited by the administration of secretin. In the paper above referred to five cases treated with secretin are mentioned. In two the results were negative, but it is stated that the periods of observation were short and the doses insufficient. In a third case, a man aged 25, after administration of the extract for some weeks the sugar fell in amount and gradually disappeared entirely. The patient increased in weight and the polyuria disappeared. Unfortunately a few months later, after leaving the infirmary and giving up his extract, he developed phthisis, the sugar reappeared, and he shortly afterwards died. Another case was that of a boy aged 7, in whom the sugar excretion gradually fell, until after five and a half weeks' treatment it was entirely absent, and remained so, even after the treatment had been suspended. In a further case, a girl aged 9 years, the sugar entirely disappeared after three weeks' treatment.

During the last few months I have treated three patients by this method, but unfortunately with entirely negative results. The cases, even though failures, are, however, I think worth recording. At present it is quite uncertain what type of patient suffering from the disease is likely to respond to the treatment, yet if both negative and positive cases be recorded, some facts may be brought to light which may make this point more clear.

**Case I.** William T., aged 41, miner. Admitted February 5th, 1906. Suffering from great thirst and hunger, weakness and loss of flesh of seven months' duration. No complications.

| DATE. | | Amount of Urine in ounces. | Total Daily Amount of Sugar in grains. | Urea. | Acetone. | Diacetic Acid. | Weight. |
|---|---|---|---|---|---|---|---|
| | | | | | | | st. lb. |
| February | 6 | 19+ | 26·25 grs. per oz. | 10 grs. per oz. | None. | None. | 8 0 |
| ,, | 8 | Administration of secretin began. | | | | | |
| ,, | 8 | 80 | 2,800 | 700 | — | — | — |
| ,, | 12 | 74 | 1,420 | 841 | — | — | — |
| ,, | 15 | 64 | 704 | 755 | — | — | — |
| | | Secretin omitted from 15th—17th. | | | | | |
| ,, | 17 | 60 | 570 | 864 | — | — | 8 1 |
| ,, | 20 | 48 | 734 | 691 | — | — | |
| ,, | 25 | 56 | 852 | 1,015 | — | — | 8 1 |
| March | 1 | 60 | 780 | 929 | — | — | |
| ,, | 5 | 60 | 783 | 738 | Trace. | — | 8 1 |
| ,, | 7 | 64 | 832 | 612 | — | — | |
| ,, | 12 | 54 | 702 | 713 | — | — | 8 2 |
| ,, | 16 | 74 | 1,296 | 1,036 | — | — | |
| ,, | 18 | 62 | 920 | 988 | — | — | 8 2 |
| | | Secretin omitted. | | | | | |
| ,, | 22 | 78 | 1,014 | 12,48 | — | — | — |
| ,, | 26 | 74 | 1,295 | 10,36 | — | — | 8 2 |

**Case II.** George K, aged 27, gardener. Admitted March 3rd, 1906, for thirst and polyuria of eighteen months' duration, with loss of flesh. There was no complication.

| DATE. | | Amount of Urine in ounces. | Daily Amount of Sugar in grains. | Urea. | Acetone. | Diacetic Acid. | Weight. |
|---|---|---|---|---|---|---|---|
| | | | | | | | st. lb. |
| March | 4 | 64 | 2,240 | — | Absent. | Absent. | 10 4 |
| ,, | 6 | 52 | 840 | 765 | — | — | — |
| ,, | 8 | 48 | 704 | 701 | — | — | — |
| ,, | 11 | 48 | 420 | 764 | — | — | — |
| ,, | 13 | 64 | 830 | 1,066 | — | — | 10 1 |
| ,, | 17 | 58 | 1,270 | 1,160 | Absent. | Absent. | 10 1 |
| ,, | 19 | Administration of secretin began. | | | | | |
| ,, | 19 | 74 | 1,295 | 1,180 | — | — | — |
| ,, | 21 | 62 | 1,170 | 1,302 | — | — | — |
| ,, | 26 | 74 | 888 | 1,406 | — | — | 10 2 |
| ,, | 30 | 72 | 950 | 1,260 | — | — | — |
| April | 1 | 72 | 1,160 | — | — | — | — |
| ,, | 5 | 65 | 975 | 1,278 | — | — | 10 1 |
| ,, | 9 | 68 | 1,020 | 1,156 | — | — | — |
| ,, | 13 | 70 | 910 | 1,330 | — | — | — |
| ,, | 16 | Secretin solution stopped. | | | | | |
| ,, | 18 | "Antiglucosine" substituted. | | | | | |
| ,, | 18 | 58 | 870 | 989 | — | — | — |
| ,, | 22 | 74 | 806 | 1,358 | — | — | 10 2 |
| ,, | 26 | 58 | 826 | 1,282 | — | — | — |
| ,, | 30 | 58 | 754 | 913 | — | — | — |
| May | 4 | 60 | 780 | 1,181 | — | — | 10 2 |
| ,, | 9 | 60 | 780 | 1,050 | — | — | — |
| ,, | 21 | 52 | 676 | 884 | — | — | — |
| ,, | 25 | 59 | 826 | 12,84 | — | — | — |
| June | 5 | 56 | 980 | 840 | — | — | 10 2½ |
| ,, | 11 | 48 | 720 | 802 | — | — | — |
| ,, | 13 | 60 | 780 | 1,140 | — | — | — |

**Case III.** John W., aged 53, shopkeeper. Admitted November 16th, 1905, and discharged January 16th, 1906. Complained of thirst, excessive appetite, polyuria, dryness of skin, and loss of weight of three and a half years' duration. This man was treated by diet, Codeia and Aspirin. He went out unrelieved. A quantitative and qualitative investigation of his urine was made twice a week. The analyses remained remarkably constant. Acetonuria was present throughout. Comparing the last with the first analysis we have:—

| DATE. | Oz. | Sugar. | Urea. | Acetone. | Diacetic Acid. | Weight. |
|---|---|---|---|---|---|---|
| | | | | | | st. lb. |
| November 20 | 84 | 2,499 | 882 | Present. | Absent. | 8 10 |
| January 15 | 104 | 2,631 | 676 | Present. | Present. | 9 0½ |

Since his discharge he has been kept under constant observation, and on February 6th secretin treatment was commenced. As the daily amount of urine was only roughly estimated, the amount of sugar was calculated by grains per ounce. The following figures show that little improvement took place. Acetone disappeared on May 15th, but rapidly returned:—

| DATE. | Grains per oz. | DATE. | Grains per oz. |
|---|---|---|---|
| February 13 | 28·3 | April 20 | Began "Antiglucosine." |
| ,, 27 | 28·9 | May 8 | 26·2 |
| March 3 | 26·8 | ,, 15 | 29·2 |
| ,, 9 | 28·6 | ,, 21 | 21·4 |
| ,, 16 | 31·4 | June 11 | 39·2 |
| ,, 23 | 25.7 | ,, 21 | Stopped "Antiglucosine." |
| ,, 30 | 32·2 | | |

The "Antiglucosine" mentioned above is an acid extract of duodenal mucous membrane, and differs from the solution of secretin first used in being made freshly every other day. Solutions of secretin are known to become inert on keeping, and it was thought that the negative results obtained with the first solution of secretin might be due to the fact that it had been kept too long, and hence "Antiglucosine" was substituted. The treatment has for the present been discontinued, as it was found that the solution was liable to undergo decomposition on very hot days.

The most satisfactory results obtained by Moore, Edie, and Abram were in two children, whereas these patients were all adults. The diet while the observations were being made was, of course, kept constant.

